# Isoleucine at position 137 of haemagglutinin acts as a mammalian adaptation marker of H9N2 avian influenza virus

**DOI:** 10.1080/22221751.2025.2455597

**Published:** 2025-01-16

**Authors:** Weiwei Ma, Chenyang Ren, Lin Shi, Bo Meng, Yali Feng, Ying Zhang

**Affiliations:** a Key Laboratory of Livestock Infectious Diseases, Ministry of Education, Key Laboratory of Zoonosis, College of Animal Science and Veterinary Medicine, Liaoning Panjin Wetland Ecosystem National Observation and Research Station, Shenyang Agricultural University, Shenyang, People’s Republic of China; b Poultry Diseases Research Laboratory, Liaoning Center for Prevention and Control of Animal Infectious Diseases, Shenyang, People’s Republic of China

**Keywords:** Avian influenza virus, H9N2, haemagglutinin, receptor-binding property, stability

## Abstract

The H9N2 subtype of avian influenza virus (AIV) is widely distributed among poultry and wild birds and is also a threat to humans. During AIV active surveillance in Liaoning province from 2015 to 2016, we identified 10 H9N2 strains exhibiting different lethality to chick embryos. Two representative strains, A/chicken/China/LN07/2016 (CKLN/07) and A/chicken/China/LN17/2016 (CKLN/17), with similar genomic background but different chick embryo lethality, were chosen to evaluate the molecular basis for this difference. A series of reassortants between CKLN/07 and CKLN/17 were generated and their chick embryo lethality was assessed. We found that the isoleucine (I) residue at position 137 (H3 numbering) in the haemagglutinin (HA) was responsible for the chick embryo lethality of the H9N2 virus. Further studies revealed that the threonine (T) to I mutation at HA position 137 enhanced viral replication in vitro and in vivo. Moreover, the HA-T137I substitution in H9N2 avian influenza virus increased the guinea pig transmission efficiency. We also found that the HA-T137I substitution was critical for α2,6-linked sialic acid binding preference and HA activation and stability of H9N2 virus. Our findings demonstrated that HA-137I is a key molecular marker for mammalian adaptation of H9N2 AIV.

## Introduction

Avian influenza virus (AIV) is an enveloped Orthomyxoviridae family virus with a segmented, single-stranded, negative-sense RNA genome. The H9N2 AIV first emerged in Guangdong province, China, in 1992. During the outbreaks of H9N2 AIV from 1992 to 1994 in China, the mortality rate of broiler chickens ranged from 10% to 40%, and egg production decreased by 14–75%, however, when inoculated SPF chickens with the outbreak strains, only mild flu-like symptoms could be observed [1]. In 1996, Korea reported H9N2 AIV outbreaks and indicated that serially passaged the H9N2 AIVs in chicken embryo eggs could significantly increase the per cent mortality in embryos and pathogenicity in chicken [2]. After the outbreak, the H9N2 AIVs gradually became the dominant subtype in poultry [3]. In addition to poultry, H9N2 AIVs can infect a broad spectrum of wild birds and various mammals, such as pigs, dogs, minks, civets, Asian badgers, and even humans [1, 4, 5]. As of May 2024, the H9N2 AIVs have resulted in 128 human infections, including two deaths (https://www.canada.ca/en.html), indicating a threat to public health.

Studies have shown that H9N2 AIVs can replicate in mice without pre-adaptation and some strains can transmit between ferrets through contact or respiratory droplets [6–8]. Mechanistic studies indicate that the acquisition of the ability to bind to human-type receptors (α2,6-linked sialic acid) is critical for H9N2 AIV to adapt to mammalian hosts. Several amino acid substitutions in the HA protein have been confirmed to enhance the human receptor-binding affinity of H9N2 AIVs, including isoleucine (I) to threonine (T) at position 155, alanine (A) to aspartic acid (D) or asparagine (N) at position 160, T to proline (P) at position 187, D to glutamic acid (E) at position 208, glutamine (Q) to L at position 226, methionine (M) to L at position 227, and arginine (R) to lysine (K) at position 246, thereby increasing the viral replication or pathogenicity in mammalian cells or hosts [7–12]. Synergy between HA and other viral gene segments has also been detected in H9N2 AIV mammalian adaptation (e.g. HA-T187P/M227L combined with PB2-E627K, HA-A190V or T212I with PB2-G685R, HA-Q227P/D375E with NP-E434K, and HA-A316S with the NA short-stalk mutation). These combinations not only improve H9N2 virus replication in mammalian cells but also facilitate virus transmissibility among guinea pigs [11, 13–15]. Amino acid substitutions in other gene segments have also been shown to be critical for H9N2 AIVs mammalian adaptation, including PB2 S155N and PA S49Y/D347G, PB2-Q591K, PB2-E627K, PB2-D701N, PA-K356R and PA-T97I [16–20]. Recently, the mammalian-adaptation substitution PB2-E627K was been found to be prevalent in H9N2 AIVs isolated from wild birds [21], indicating the need for increased surveillance.

Due to its low pathogenicity, H9N2 AIV prevention and control is often under appreciated. Although H9N2 virus infections alone do not induce obvious clinical signs or deaths in chickens [22], the economic losses to the poultry industry cannot be ignored. H9N2 AIV infection not only reduces poultry productivity, but also increases susceptibility to secondary infections [23]. Vaccination has, so far, been an effective strategy for AIVs control, as long as the vaccine strain is updated when an antigenically different emerging virus begins to be prevalent [24, 25]. However, some of the H9N2 influenza vaccines cannot provide solid protection to chickens against currently circulating H9N2 virus, and an effective vaccine update strategy should be established for H9N2 vaccines [23].

Because influenza virus is a genome-segmented virus, new reassortants could be generated by genome segments swapping between different influenza strains. The internal gene cassette of H9N2 AIV has been detected in H1N1, H1N2 [26], H3N8 [27], H7N2, H7N9 [28–30], H10N3, H10N8 [31, 32], H5N1, and H5N6 [33, 34] viruses, and some of these emerging reassortants have caused human infections or deaths. Studies have indicated that the internal gene cassette of H9N2 could enhance the mammalian virulence and adaptability of reassortants [35, 36]. So the H9N2 viruses could act as a “coach”, training avian-origin influenza viruses to adapt to mammalian hosts.

During active surveillance of AIVs in Liaoning Province from 2015 to 2016, we isolated ten strains of H9N2 viruses with varying lethality to chick embryos. We selected two strains, A/chicken/China/LN07/2016 (CKLN/07) and A/chicken/China/LN17/2016 (CKLN/17), which have a similar genetic background but exhibit distinct phenotypes, as represent strains to explore the molecular determinant of their pathogenicity difference. We identified a key amino acid in HA that determines the different chick embryo lethality between these viruses and explored its contribution to the mammalian host adaptation of H9N2 virus.

## Materials and methods

### Cells and viruses

Madin-Darby canine kidney (MDCK), human embryonic kidney (HEK) 293 T, chicken embryo fibroblasts UMNSAH/DF-1 (DF-1), African green monkey kidney (Vero) and Human lung adenocarcinoma epithelial (A549) cells were maintained in Dulbecco’s modified Eagle’s medium (DMEM). MDCK cells were supplemented with 5% fetal bovine serum (FBS) (Cellmax, Beijing, China), DF-1, Vero, A549, and HEK293 T cells were supplemented with 10% FBS. Primary chicken embryo fibroblasts (CEFs) were prepared from 9-day-old specific pathogen-free (SPF) embryonated eggs, and maintained with 6% FBS in Medium 199.

The H9N2 influenza viruses investigated in this study were isolated during 2015–2016 active surveillance and the whole-genome sequencing and phylogenetic analyses were conducted (Supplementary information). 10⁶ 50% tissue culture infectious doses (TCID_50_) of each virus were inoculated into five 9-day-old SPF embryonated eggs in a volume of 100 µL. The survival amounts of the inoculated eggs were checked every 12 h until 72 h post-infection (p.i.). Two strains, A/chicken/Liaoning/17/2016 (CKLN/17) and A/chicken/Liaoning/07/2016 (CKLN/07), with similar genetic characteristics but different lethality to chick embryos. We selected these two viruses as representative viruses to further study factors related to differences in pathogenicity. The gene bank access numbers of CKLN/17 and CKLN/07 are MW255950-MW255957 and MW255942-MW255949, respectively.

The eight gene segments of CKLN/07 and CKLN/17 were inserted into the pBD vector (vRNA-mRNA bidirectional transcription vector) to generate the reverse genetics system (RGS) for the two viruses. Then, a series of reassortants and mutants of CKLN/17 and CKLN/07 were rescued by single gene substitution and site-directed mutagenesis with the RGS, as described previously [37]. The whole genome of all viruses was sequenced to ensure the absence of unwanted mutations.

### Viral growth kinetics in vitro

MDCK, DF-1, and CEF cells were infected with CKLN/07, CKLN/17, and the mutants at a multiplicity of infection (MOI) of 0.001, while A549 cells were infected with these viruses at an MOI of 0.1, to compare their replicative ability. After a 1-h incubation, the cells were washed three times with PBS and overlaid with DMEM containing 1 μg/mL TPCK-treated trypsin and maintained at 37°C. Supernatants were collected at 12, 24, 36, 48, 60, and 72 h p.i. for titration.

### Viral replication in mice

Eight 6-week-old female BALB/c mice (Changsheng Biotechnology, Liaoning, China) were intranasally infected with a 50-μL volume containing 10^6^ EID_50_ of CKLN/07, CKLN/17, and their mutants. On Day 3 post-inoculation, three mice were euthanized, and their brain, nasal turbinates, lung, spleen, and kidney were collected and stored at −80°C. Virus titres were determined in 10-day-old chick embryos. The rest of mice were weighed every day until 14 days p.i..

### Guinea pig transmission study

Groups of three female Hartley strain guinea pigs, weight 300–350 g (Changsheng biotechnology, Liaoning, China), were intranasally inoculated with a 300-μL volume containing 10^6^ EID_50_ of CKLN/07, CKLN/17, and their mutants as inoculated groups. One day after inoculation, three naïve guinea pigs were placed in the same cages as the contacted groups, and the other three naïve guinea pigs were placed in the adjacent cage, located 4 cm away from the inoculated guinea pigs cage, as exposed groups. Nasal washes were collected from all the guinea pigs every other day from Day 2 post-inoculation for viral titration. On Day 21 post-inoculation, serum samples were collected from all the guinea pigs for antibody detection.

### Receptor binding assays

The receptor-binding properties of the isolates were determined by using HA assays with 1% chicken red blood cells (cRBCs) and sheep red blood cells (sRBCs) as described before [38]. The surface of cRBCs contains both α2,3-linked sialic acid and α2,6-linked sialic acid receptors, whereas the surface of sRBCs only contains α2,3-linked sialic acid receptors. For this study, cRBCs were incubated with α2,3-Sialidase (Takara, Dalian, Liaoning, China) at 37°C for 1 h to prepare cRBCs containing only α2,6-linked sialic acid receptor. As a negative control, the cRBCs were treated with Vibrio cholerae neuraminidase (Merck, Darmstadt, Germany) to remove both α2,3-linked sialic acid and α2,6-linked sialic acid. The treated cRBCs were washed with PBS and adjusted to a final working concentration (1%) with PBS for testing the receptor-binding properties of the isolates. The A/chicken/Guangxi/9/99 (CKGX/9) virus and the A/Hong Kong/4801/2014 (HK/4801), which bind to α2,3-cRBCs and α2,6-cRBCs, respectively, were used as controls [7].

The receptor binding specificity of the H9N2 viruses was also determined by using a solid-phase direct binding ELISA assay, with two glycopolymers: α2,3-sialylglycopolymer (Neu5Acα2-3Galβ1-4GlcNAc-PEG3-biotin, 3SLN) and α2,6-sialylglycopolymer (Neu5Acα2-6Galβ1-4GlcNAc-PEG3-biotin, 6SLN) (Sussex Research Laboratories Inc., Ottawa, Canada) [39]. In this study, antibody against H9N2 HA (SinoBiological, Beijing, China) was used as the primary antibody. The Horseradish peroxidase (HRP)-labeled Goat Anti-Rabbit IgG(H + L) (Beyotime, Shanghai, China) was used as the secondary antibody. The A/swine/Jiangxi/261/2016 (SWJX/261) and A/chicken/Chongqing/SD001/2021 (CKCQ/SD001) [40, 41], which bind to α2,6-linked sialic acid and α2,3-linked sialic acid, respectively, were used as controls. All viruses were inactivated with β-propiolactone (Solarbio, Beijing, China) prior to the assays. For the controls, chicken antiserum was used as the primary antibody, and HRP-conjugated goat-anti-chicken antibody (Sangon, Shanghai, China) was used as the secondary antibody. The absorbance was determined at a wavelength of 450 nm.

### Syncytium formation assay

The syncytium formation assay, as previously described, was used to determine the pH of HA activation [42]. In brief, MDCK or Vero cells were cultured in 12-well plates and infected with viruses at a MOI of 10 for MDCK and MOI of 3 for Vero cells. At 16 h post-infection, the MDCK or Vero cells were treated with 5 μg/mL of TPCK-treated trypsin for 15 min and incubated in prewarmed MES buffer with pH adjusted from 5.0 to 6.0, increasing in increments of 0.1. Subsequently, after washing the cells three times with PBS, the low-pH MES buffer was replaced by DMEM with 5% FBS for MDCK cells or 10% FBS for Vero cells, and the cells were incubated at 37°C for 3 h to allow syncytium formation. After incubation, the MDCK or Vero cells were fixed with cell fixation solution, 4% paraformaldehyde, and stained using a rabbit anti-influenza A virus NP antibody (CUSABIO, Wuhan, China) and an Alexa Fluor 488-labeled Goat Anti-Rabbit IgG (H + L) as the secondary antibody (Beyotime Biotechnology, Shanghai, China).

### HA thermostability and acid stability assays

For the viral thermostability assay, CKLN/07, CKLN/17, and their HA mutants were first diluted into 10^6.5^ TCID_50_ in PBS then incubated at 56°C for 6 h. The titres of CKLN/07, CKLN/17, and the mutants were evaluated every hour.

For the viral acid stability assay, each virus was diluted to 10^6.5^ TCID_50_ with a series of pH-adjusted PBS and incubated at 37°C for 1 h. The titres of these viruses were subsequently determined by TCID_50_ in MDCK cells.

### Statistical analysis

The data were analysed by using a two-way analysis of variance (ANOVA) in GraphPad Prism version 8.0 (GraphPad Software Inc., CA, USA). Probability (*P*) values < 0.05 were considered statistically significant, whereas *P* values < 0.01 were considered highly significant.

### Analysis the polymorphism of amino acid at position 137 in HA of H9N2 viruses in nature

HA sequences of H9N2 viruses isolated in China from 1966 to 2023 were downloaded from three databases, the National Center for Biotechnology Information (NCBI) Influenza Virus Resource (https://www.ncbi.nlm.nih.gov/genomes/FLU/Database/nph-select.cgi), the Global Initiative on Sharing All Influenza Data (GISAID) EpiFlu database (https://platform.gisaid.org/), and the Bacterial and Viral Bioinformatics Resource Center (BV-BRC, https://www.bv-brc.org/). Multiple-sequence alignments were conducted using MEGA 7 software (Pennsylvania State University, PA, USA). After removing duplicates and sequences lacking the amino acid residue at position 137, 12,469 sequences were selected. The polymorphism at residue 137 was evaluated using the “Statistics-Generate Alignment Profile” feature in UGENE software (Unipro, Novosibirsk, Russia).

### Ethics statement

The use of mice, guinea pigs, and chickens in this study followed the recommendations in the Guide for the Care and Use of Laboratory Animals of the Ministry of Science and Technology of the People’s Republic of China. The animal research was approved by the Committee on the Ethics of Animal Experiments of the Key Laboratory of Livestock Infectious Diseases in Northeast China, Ministry of Education (Approval No. 202106009 and No. 24082901, granted on March 8, 2021 and August 29, 2024.).

## Results

### Isoleucine at position 137 in the HA protein determines chick embryo lethality

**Figure 1.**
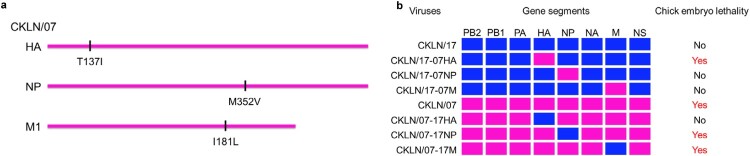
(a) Amino acid variations between the two H9N2 avian influenza viruses. The amino acid differences between CKLN/07 and CKLN/17 are shown as single letters at the indicated positions. Each amino acid of CKLN/17 is shown before the number of the position, and each amino acid of CKLN/07 is shown after the number of the position. (c) Viral lethality to embryonated chicken eggs was evaluated using a reverse genetics system for single gene substitution, measuring mortality within 72 hpi with SPF chicken embryos.

The ten H9N2 strains isolated in our surveillance have similar genetic background and their genome segments were clustered into the same branch in the phylogenetic trees (Supplementary information, Figure S1a-1h). However, these strains exhibited varying degrees of lethality in chick embryos (Table S1). Death began to appear in the CKLN/07 inoculated embryonated eggs since 42 h p.i. The CKLN/07 inoculated eggs all died in 72 h p.i., but the CKLN/17 inoculated eggs all survived (Figure S2). Sequence analysis identified three amino acid differences between CKLN/07 and CKLN/17 in their HA, NP, and M proteins (Figure 1(a)). To explore the gene responsible for the difference in chick embryo lethality, we generated RGSs by swapping the HA, NP, and M genes between CKLN/07 and CKLN/17. Reassortants based on the CKLN/07 backbone were designated CKLN/07-17HA, CKLN/07-17NP, and CKLN/07-17M, while those based on the CKLN/17 backbone were designated CKLN/17-07HA, CKLN/17-07NP, and CKLN/17-07M. Evaluation of chick embryo lethality showed that the M and NP genes didn’t influence the virulence of the H9N2 viruses. But when the HA gene of the two viruses was swapped, the CKLN/07-17HA could not kill chick embryos, whereas CKLN/17-07HA exhibited lethality in chick embryos (Figure 1(b)). The only difference in the HA proteins of CKLN/07 and CKLN/17 was the amino acid at position 137 (H3 numbering), showing that HA-137I plays a key role in chick embryo lethality. Furthermore, the 50% egg lethal dose (ELD_50_) of CKLN/07, CKLN/17, CKLN/07-HA-I137 T, and CKLN/17-HA-T137I were evaluated (Supplementary information). CKLN/17 acquired similar chick embryo lethality as CKLN/07 after the HA-T137I substitution (Table S2).

### The HA-T137I substitution increases the replication of H9N2 virus in different sources of cells

**Figure 2.**
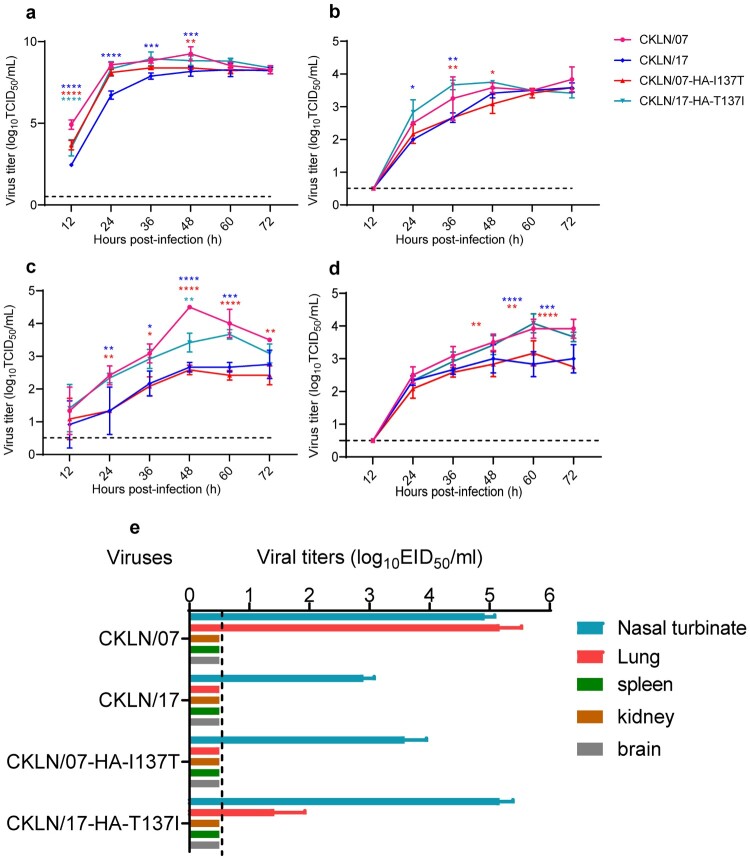
The viral replication kinetics of H9N2 viruses in MDCK (a), CEF (b), DF-1 (c) and A549 (d) cells. A549 cells were infected in triplicate with the test viruses at a MOI of 0.1 and other cells were infected at a MOI of 0.001. Supernatants were collected at the indicated timepoints for titration in MDCK cells. The black dashed line represents the lower limit of detection. Significance was analysed using a two-way ANOVA and compared with CKLN/07. (e) Replicative ability of H9N2 viruses in mice. Groups of mice were infected with 10^6^ EID_50_ of the specified viruses in a volume of 50 μl. On the 3 dpi, changes in body weight were recorded, and six organs were collected and titrated in eggs. Except for the lungs and turbinates, no viral titres were detected in other organs. The mice did not show any weight loss at the end of the experiment. The titration was calculated using the Reed-Muench method and expressed as the mean log_10_ EID_50_/mL ± SD. For statistical purposes, the value of virus titration-negative samples was set to 0.5., *P < 0.05; **P < 0.01; ***, *P* < 0.001; ****P < 0.0001.

The replicative ability of CKLN/07, CKLN/17, and their HA mutants were compared in MDCK, DF-1, CEF, and A549 cells. CKLN/07 exhibited significantly higher replication than CKLN/17 in all four kinds of cells (Figure 2(a–d)). Introducing the HA-T137I substitution into CKLN/17 enhanced its viral replication, even in CEF cells the viral titre of CKLN/17-HA-T137I were significantly higher than CKLN/07; conversely, the HA-I137 T substitution in CKLN/07 reduced its viral replication capacity. These findings indicate that 137I in the HA protein H9N2 AIVs enhances viral replication in not only mammalian cells but also avian cells.

### The HA-T137I substitution enhances the replication of H9N2 virus in mice

The replications of CKLN/07, CKLN/17, and the two HA mutants in one-day-old chickens were detected in trachea and lung, and no significant differences were observed among the infected groups (Figure S3). We then assessed the replication of those strains in mice (Figure 2(d)). The replication of the four viruses was restricted to mouse nasal turbinates or lung. CKLN/17 exhibited lower replicative ability than CKLN/07, and could only be detected in the nasal turbinate of the inoculated mice. After the acquisition of the HA-T137I substitution, CKLN/17-HA-T137I could replicate in mouse lung and the viral titre in the nasal turbinate increased nearly 100 times. In contrast, the replication of CKLN/07-HA-I137T was suppressed; the virus could not replicate in mouse lung and the viral titre in the nasal turbinates decreased 10 times compared with CKLN/07. No clinical symptoms were observed from all the infected mice, all mice increased bodyweight at the end of the experiment. Mild pathological change, thicken alveolar walls, slight alveoli collapse were observed in CKLN/07 and CKLN/17-HA-T137I infected mice lung (Figure S4c, S4f). The 50% mouse lethal dose (MLD_50_) of CKLN/07, CKLN/17, CKLN/07-HA-I137T, and CKLN/17-HA-T137I were determined by inoculating groups of five 6-week-old female BALB/c mice (Supplementary information, Table S2). These results indicated CKLN/07 and CKLN/17 were low pathogenic in mice, and the HA-137 mutations wouldn’t alert their pathogenicity.

### The HA-T137I substitution enhances the transmission of H9N2 virus among guinea pigs

**Figure 3.**
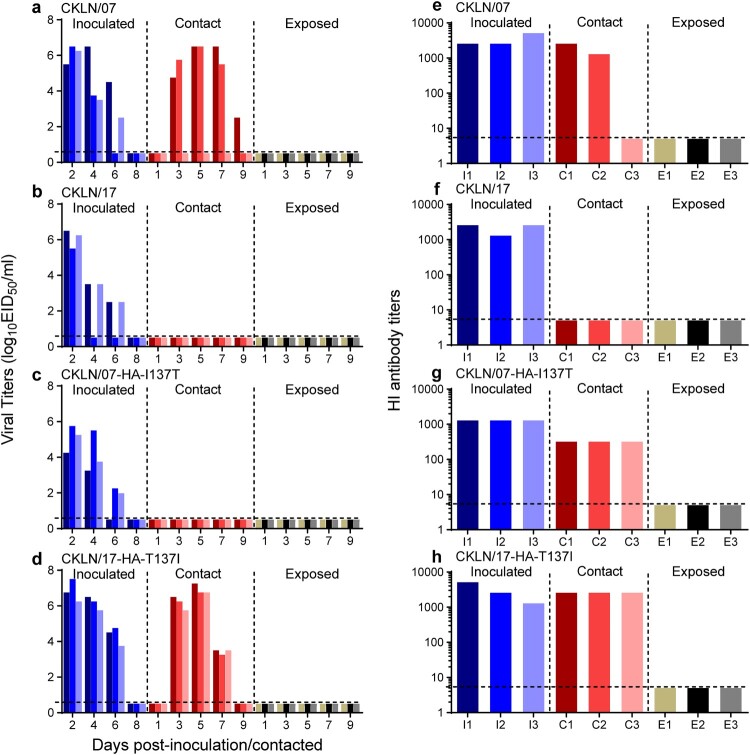
Transmission of the two H9N2 strains and their mutants in guinea pigs. Groups of guinea pigs were infected with 10^6^ EID_50_ of viruses in a volume of 300 μl. At 24 h after infection, three naive guinea pigs were introduced into the cage with the inoculated guinea pigs, while another three naive guinea pigs were placed in a neighbouring cage. Nasal washes were collected at the designated timepoints and titrated in embryonated eggs. The black dashed lines indicate the limit of titre detection. Each colour bar represents the virus titre from an individual animal. (a) CKLN/07 viral titres, (b) CKLN/17 viral titres, (c) CKLN/07-HA-I137 T viral titres, (d) CKLN/17-HA-T137I viral titres, (e) CKLN/07 HI antibody titres, (f) CKLN/17 HI antibody titres, (g) CKLN/07-HA-I137 T HI antibody titres, (h) CKLN/17-HA-T137I HI antibody titres.

We used the guinea pig transmission model to assess the impact of the HA-T137I substitution on the transmissibility of H9N2 virus. No virus was detected from any exposed guinea pigs, or CKLN/17-, or CKLN/07-HA-I137T-contact guinea pigs. However, transmission occurred in contact guinea pigs in the CKLN/07 group (Figure 3(a)). Virus could be detected in two contact guinea pigs from Day 3 post-inoculation, and on Day 9 post-inoculation, virus could be detected in only one guinea pig. When changed the CKLN/17-HA 137 amino acid from T to I, efficient transmission occurred in the CKLN/17-HA-T137I-contact group guinea pigs. Virus was detected in all three contact guinea pigs from Day 3 to Day 7 post-inoculation (Figure 3(d)). When the HA-I137T substitution was introduced into CKLN/07, the opposite result was observed in the CKLN/07-HA-I137T group (Figure 3(c)). Seroconversion occurred in all of the virus-inoculated and in all contacted guinea pigs that were virus-positive. Even though no virus was detected in the contact animals from the CKLN/07-HA-I137T group, antibodies were detected in these guinea pigs (Figure 3(e–h)). Probably, the limited amount of CKLN/07-HA-I137 T virus did transmit to the contact animals and stimulate their immune response but the virus didn’t establish an effective infection. These results confirm the contribution of HA 137I to the transmission efficiency of H9N2 virus.

### The amino acid at HA 137 affects the receptor binding preference of H9N2 virus

**Figure 4.**
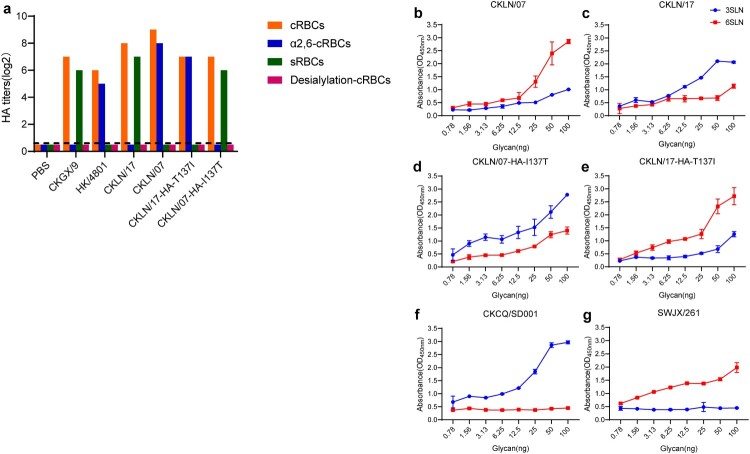
Receptor-binding specificity of the H9N2 viruses. (a) The receptor-binding specificity of CKLN/07 and CKLN/17 was assessed using cRBCs without α2,3-linked sialic acid and sRBCs in an HA assay. Each coloured bar corresponds to the haemagglutination titre. (b–g) Binding of the indicated viruses to sialylglycopolymers (α2,3-linked sialic acid, red; α2,6-linked sialic acid, blue). The data shown are the means of three repeats; the error bars indicate standard deviations.

As HA receptor-binding specificity significantly impacts the transmission of influenza viruses, we analysed receptor binding by using cRBCs without α2,3-linked sialic acid and sRBCs to examine the receptor-binding specificity of CKLN/07 and CKLN/17. We prepared chicken and sheep erythrocytes as described previously [43] and conducted haemagglutination assays. We found that CKLN/07 and CKLN/17-HA-T137I could bind to α2,6-cRBCs the same as HK/4801, whereas CKLN/17 and CKLN/07-HA-I137T only bound to sRBCs like CKGX/9 (Figure 4(a)). Solid-phase binding analysis indicated that CKLN/07 and CKLN/17-HA-T137I bound to α−2, 6-sialylglycopolymer with high affinity and to α−2, 3-sialylglycopolymer with low affinity while CKLN/17 and CKLN/07-HA-I137T represented the opposite binding affinity preference. (Figure 4(b–e)). These results indicate that HA-137I plays an important role in H9N2 influenza virus binding preference to α2,6-linked sialic acid receptors, which facilitates the mammalian transmission of the virus.

### The HA-T137I substitution reduces the activation pH of H9N2 virus

**Figure 5.**
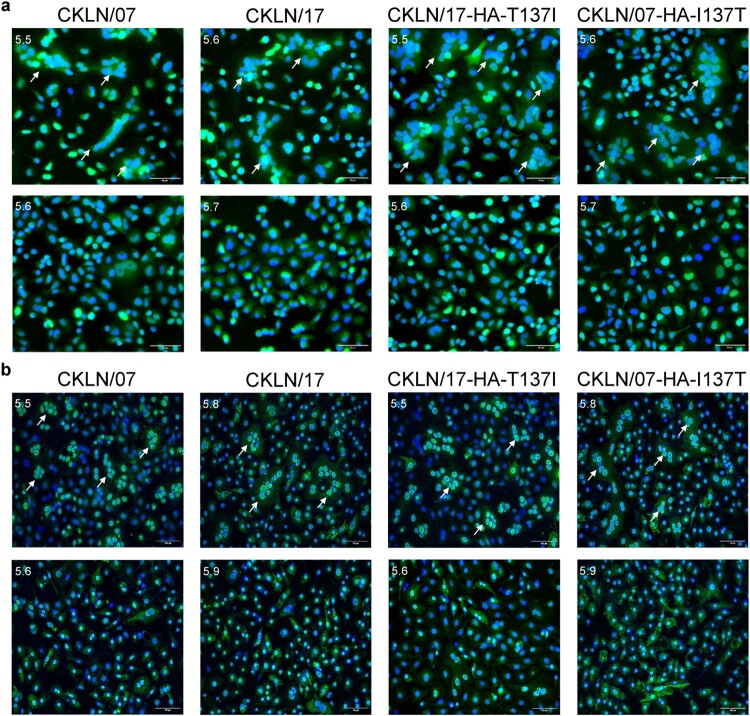
The impact of pH on HA activation of the H9N2 viruses was examined in MDCK (a) and Vero (b) cells. The highest pH at which syncytia formation (indicated by an arrow) exceeded 50% was determined as the pH threshold.

The HA protein of influenza virus is activated at low pH to change its confirmation and initiate the fusion process. Lower pH of HA activation has been shown to be correlated with mammalian adaptation of IAVs [44, 45]. The pH of HA protein activation can be measured by using the syncytium formation assay. By using this assay, we found that the pH of HA activation of CKLN/07 and CKLN/17-HA-T137I (pH 5.5) was lower than that of CKLN/17 and CKLN/07-HA-I137T (pH 5.6) in MDCK cells (Figure 5(a)). In Vero cells, HA activation threshold of CKLN/07 and CKLN/17-HA-T137I was at pH 5.5, while the threshold of CKLN/17 and CKLN/07-HA-I137T was at pH 5.8 (Figure 5(b)). These results indicate that the T137I substitution in HA reduces the pH of HA activation in different mammalian cells.

### The HA-T137I substitution enhances the thermostability but decreases the acid stability of H9N2 virus

**Figure 6.**
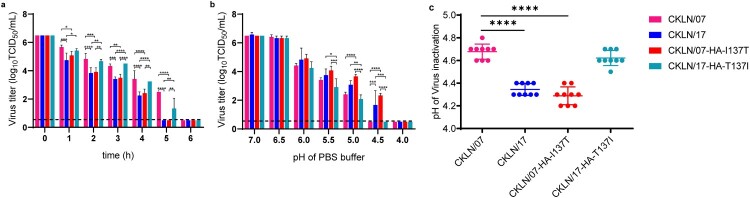
The stability of H9N2 avian influenza viruses. (a) Thermostability of H9N2 viruses. 10^6.5^ TCID_50_ of each virus was incubated at 56°C for 6 h, and virus titres were determined in MDCK cells hourly. (b) Acid stability of H9N2 viruses. 10^6.5^ TCID_50_ of each virus was diluted in PBS adjusted to the indicated pH and incubated at 37°C for 1 h. Virus titres were then determined in MDCK cells. (c) Virus inactivation pH values. Viruses were treated with pH-adjusted PBS for 1 h, then neutralized with 0.001 M NaOH, and infectivity was assessed by TCID_50_. *P < 0.05; **P < 0.01; ***P < 0.001; ****P < 0.0001.

There are correlations between influenza virus stability and viral environmental persistence, transmissibility, and host adaptation [45, 46]. To investigate the impact of the amino acid at position 137 on the stability of HA, we investigated the thermostability and acid stability of CKLN/07, CKLN/17, and their HA mutants. For the thermostability assay, we incubated 10^6.5^ TCID_50_ of each virus for 6 h at 56°C and assessed the viral titre every hour. All the viral titres decreased as the incubation time increased. The CKLN/17 and CKLN/07-HA-I137 T titres decreased faster than those of CKLN/07 and CKLN/17-HA-T137I. CKLN/17 and CKLN/07-HA-I137T lost infectivity completely by hour 5 at 56°C, whereas CKLN/07 and CKLN/17-HA-T137I infectivity lasted for 6 h at 56°C (Figure 6(a)), indicating that HA-137I contributes to the higher thermostability of H9N2 influenza virus.

For the acid stability assay, the viruses were diluted in PBS of varying pH values and incubated at 37°C for 1 h. For all tested viruses, the viral titres decreased as the pH value decreased. But the titre declines of CKLN/07 and CKLN/17-HA-T137I were greater than those of CKLN/17 and CKLN/07-HA-I137T. CKLN/07 and CKLN/17-HA-T137I lost infectivity at pH 4.5, whereas CKLN/17 and CKLN/07-HA-I137T lost infectivity at pH 4.0 (Figure 6(b)). We then measured each virus inactivated pH value and found that CKLN/07, CKLN/17, CKLN/07-HA-I137T, and CKLN/17-HA-T137I were inactivated at pH 4.7, 4.3, 4.3, and 4.6, respectively (Figure 6(c)). The results indicate that virus carrying HA-137 T has greater acid stability than HA-137I-bearing strains.

### Polymorphism at position 137 of HA protein of H9N2 influenza virus

**Figure 7.**
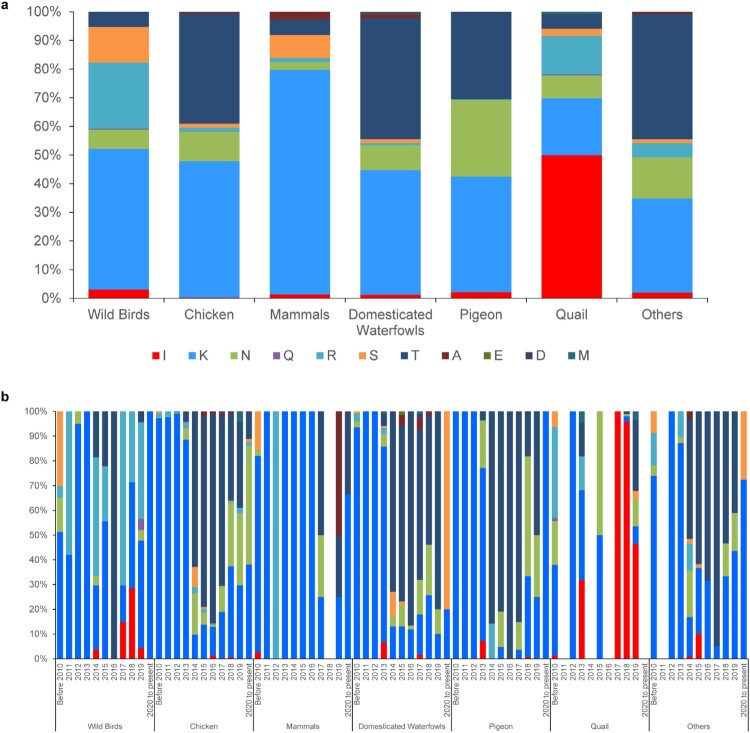
(a) The percentage of each amino acid residue at HA-137 of H9N2 influenza viruses from different hosts. (b) Historical distribution of the amino acid residue at HA-137 of various origin influenza viruses.

In order to evaluate the natural variation form of the HA-137 amino acid, we examined the polymorphism of the amino acids at this. Overall, the amino acid at this position is predominantly K across various sources, with the exception of quails (Figure 7(a)). In the chicken isolates, the amino acid at position 137 of the HA protein was predominantly K until 2013, but transitioned to T between 2014 and 2017, and then changed to N after 2018 (Figure 7(b)). Conversely, in mammalian isolates, HA-137 K remained predominant after 2020. Surprisingly, HA-137I was predominantly found in quail isolates (Figure 7(a)) and remained dominant from 2017 to 2019 (Figure 7(b)).

## Discussion

The H9N2 influenza viruses isolated during our active surveillance in Liaoning province possess similar genetic backgrounds (as shown in Figure S1) but show different chick embryo lethality. The HA gene, especially the amino acid at position 137 of HA, was found to play a key role in the chick embryo lethality difference between the two H9N2 viruses. The HA-T137I substitution enhanced the replication of the virus in mammalian cells and mice and its transmission efficiency between guinea pigs. HA-T137I substitution leads to multifunctional changes in the biological characteristics of H9N2 AIV, including receptor-binding preference, the pH of HA activation, and, viral stability.

The H9N2 viruses are considered to be low-pathogenicity AIV. However, embryonic passaging could significantly increase embryo egg pathogenicity of some H9N2 strains but with no change at the HA cleavage site [2, 47]. The H9N2 isolates in our surveillance exhibited various chicken embryo lethality. CKLN/07 caused 100% chicken embryo mortality with a mean death time as 56 h, but CKLN/17 caused no chicken embryo death (Figure S2). Our study demonstrated that the HA-137I mutation is the key molecular marker for the H9N2 chicken embryo lethality. More than that, further study indicated that the HA-137I also enhanced the H9N2 AIV replication in both avian and mammalian cells. The HA-T137I mutation seemed to have no impact on H9N2 virus replication and tissue tropism in chicken, but did enhance the replication in mice and transmission in guinea pigs. Neither CKLN/07 nor CKLN/17 caused any death in one-day-old chicken during 14 days observation period (supplementary information), when inoculated chicken with either virus in the same amount (10^6^ TCID_50_) as used in embryo mortality assessment. CKLN/07, CKLN/17, and their HA mutants replicated similarly in the respiratory system of the chicken. If we had only used the chicken model in our study, there’s no difference would be found between CKLN/07 and CKLN/17, the contribution of HA-137I to the mammalian adaptation of H9N2 AIV would not have been recognized. Therefore, we assumed that chicken embryo lethality may be used to assess the pathogenicity differences between low pathogenic AIV strains.

However, the mammalian pathogenicity of H9N2 AIV seemed to be unaffected by the HA-T137I mutation. The mice infected with CKLN/07, CKLN/17, and their HA mutants represented no clinical symptoms or bodyweight loss during the experiment time. But the HA-T137I enhanced the H9N2 replication in mice. Due to acquiring the HA-137I mutation, CKLN/17 not only increased its replication titre in the upper respiratory tract of mice but also expanded its tissue tropism.

Receptor binding specificity of influenza virus has considerable influence on replication and transmission. A switch in receptor binding specificity from α2,3-linked sialic acid to α-2,6 linked sialic acid receptors, is considered to be necessary for an avian virus to become transmissible among mammalian hosts [42, 48–52]. The receptor binding specificity changes can be acquired by specific mutations in the receptor-binding domain (RBD) of HA protein. The RBD comprises a 190-helix (186–190 aa), a 130-loop (134–138 aa), and a 220-loop (221–228 aa). Mutations in and around the RBD can influence receptor recognition. The amino acid at position 137 is located in the 130-loop and the HA-T137I substitution enhances the affinity of H9N2 AIV for α2,6-linked sialic acid receptors. The amino acid at position 137 of HA has also been reported to influence the receptor binding ability of other influenza virus subtypes: HA-137 T has been shown to impact the haemagglutination of H6N2 AIV, HA-137S facilitates H6N6 AIV binding to both avian and human receptors, and HA-137N enhances H2N2 and H3N2 AIV binding to human receptors [53, 54]. In this study we used two methods, haemagglutination assay with resialyated RBCs and solid phase direct binding test with glycopolymers, to identify the receptor biding property of H9N2 virus. Though the conclusions obtained by the two methods were similar, there were still differences. The haemagglutination assay tends to present the qualitative conclusion, while the solid phase direct binding test tends to provide the quantitative results. This indicated we could select either method for receptor binding property analysis according to the specific needs [52, 55, 56].

The genetic determinants of influenza virus transmission have not yet been clarified completely. Multiple mutations affected the receptor binding specificity or replication of influenza virus also had been proven to be associated with viral transmission [7, 57–59]. The HA-I155 T, HA-T189A, and HA-Q226L mutations enhanced the binding affinity of H9N2 AIVs to α2,6-linked sialic acid, at the same time promoted virus transmission in ferrets [7, 59, 60]. The HA-T187P and HA-M227L mutations combination raised the H9N2 AIVs replication in mammalian cells and enhanced contact viral transmission in guinea pigs [11]. Our study suggests that HA-T137I mutation can enhance the replication of H9N2 AIV in mice and switch the receptor binding specificity, at the same time render the H9N2 virus transmissible in mammals. Increased thermostability has been reported to correlate with enhanced transmissibility of AIV in mammalian [14, 42, 45]. The HA2-D46E mutation in H9N2 AIV enhanced the viral thermostability and promoted virus contact transmission in guinea pigs [14]. The HA-H107Y and HA-T318I mutations increased the thermostability of H5N1 AIV, and contributed to the H5N1 AIV aerosol transmission [61]. A lower pH environment induces a conformational change in the HA protein, leading to the release of the viral genome into the host cell cytoplasm. A premature conformational change in HA may render viruses noninfectious before they can bind to the host cell receptor [56, 62–64]. Therefore, HA acid stability has been considered associating with host adaptation and cross-species transmission [65, 66]. Human adaptation of 2009 pandemic H1N1 (pH1N1) influenza virus was reported associate with increased stability of HA protein. HA activation pH value of H1N1 Swine influenza viruses ranged from 5.5 to 6.0, while the HA activation pH value of earlier pH1N1 isolates was around 5.5. After circulation among the human population, by 2010–2012, the latter pH1N1 isolated developed higher stabilization of HA, their HA activation pH value ranged from 5.2 to 5.4 [44, 66]. The HA-T137I substitution decreased the HA activation pH from 5.8 to 5.5, which is close to the HA activation pH threshold of human influenza viruses (pH 5.0-5.5) [63]. However, the acid stability of CKLN/07 (HA-137I) was lower than that of CKLN/17 (HA-137T), as the inactivation pH of CKLN/07 was pH 4.6 and of CKLN/17 was pH 4.3. Enhanced acid stability is usually accompanied by a decreased HA activation threshold, which correlates with enhanced pathogenicity, transmissibility, or adaptation in mammalian hosts [42, 67, 68]. But this positive correlation between lower HA activation threshold and higher acid stability was not presented in our study. We found that higher acidity was not always better and that an optimal HA acidity was needed for adaptation to the mammalian host.

HA-137 amino acid polymorphism analysis indicated that quail-derived viruses predominantly exhibit “I” at this site. As the respiratory tracts of quails contain both human-like and avian-like receptors [58], quails are considered a “mixing vessel” for influenza viruses [59, 60]. There is a high possibility that the H9N2 strains with HA-137 T are more adapted to avian hosts, whereas HA-T137I H9N2 mutants acquire the substitution in quails and become adapted to mammalian hosts.

In summary, this study revealed the effect of HA-137I on chick embryo lethality and explored the mechanism for mammalian adaptation of H9N2 AIV. Considering the increasing virulence, infectivity, and transmissibility of H9N2 AIVs in mammalian species, intensified surveillance of H9N2 AIVs is essential to curb their spread and alleviate the potential impact on human health.

## Supplementary Material

Figure S3.tif

Table S1.docx

Supplementary materials.docx

Figure S2.tif

Table S2.docx

Table S3.docx

Figure S4.tif

Figure s1.docx

Figure S1.tif
