# Supplementary figures and images for: Isoleucine at position 137 of haemagglutinin acts as a mammalian adaptation marker of H9N2 avian influenza virus
Source: Emerg Microbes Infect. 2025 Jan 16;14(1):2455597. doi: 10.1080/22221751.2025.2455597 (PMC11789229; doi:10.1080/22221751.2025.2455597)

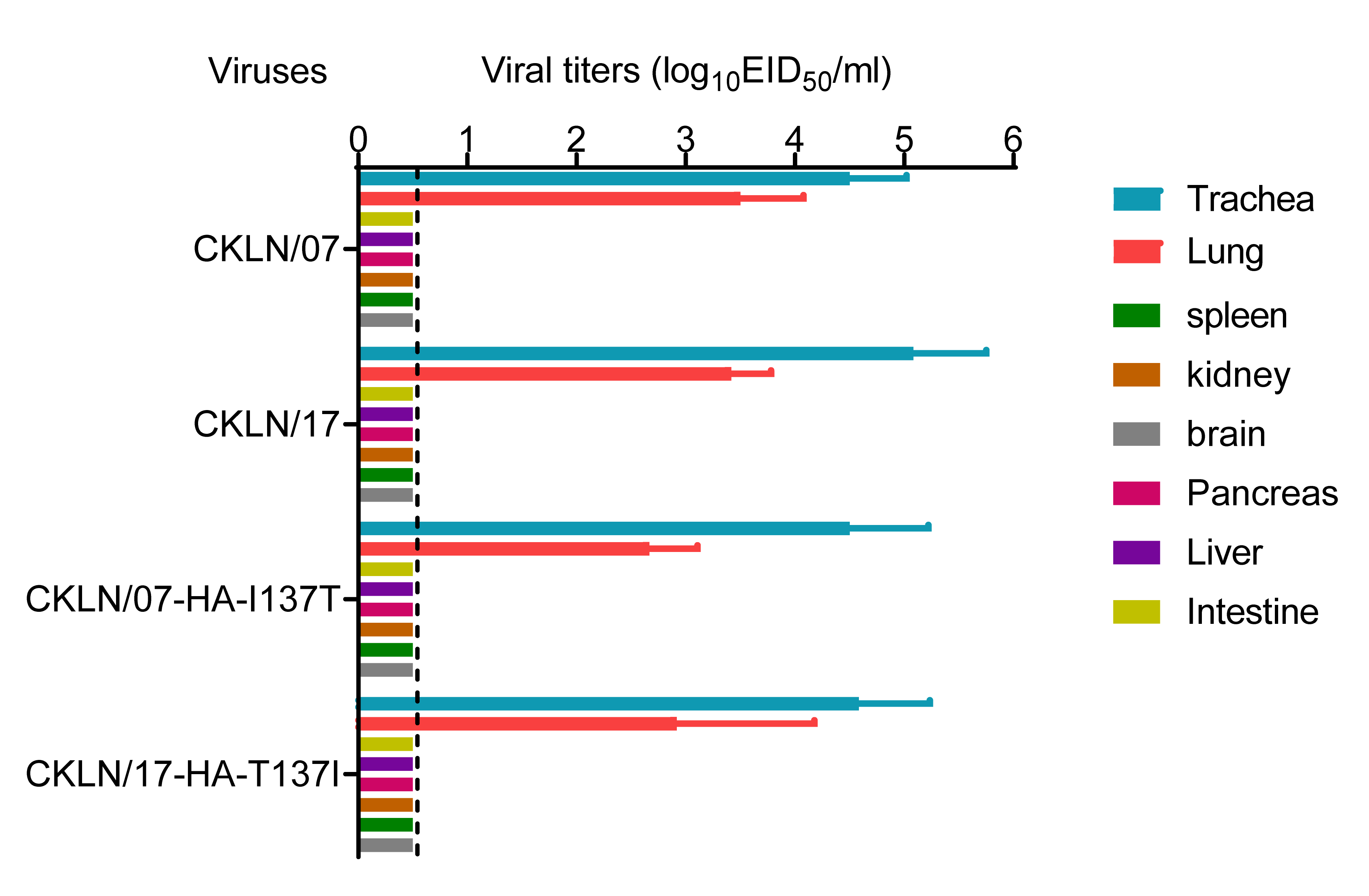

Supplement: Figure S3.tif [file TEMI_A_2455597_SM8471.tif]

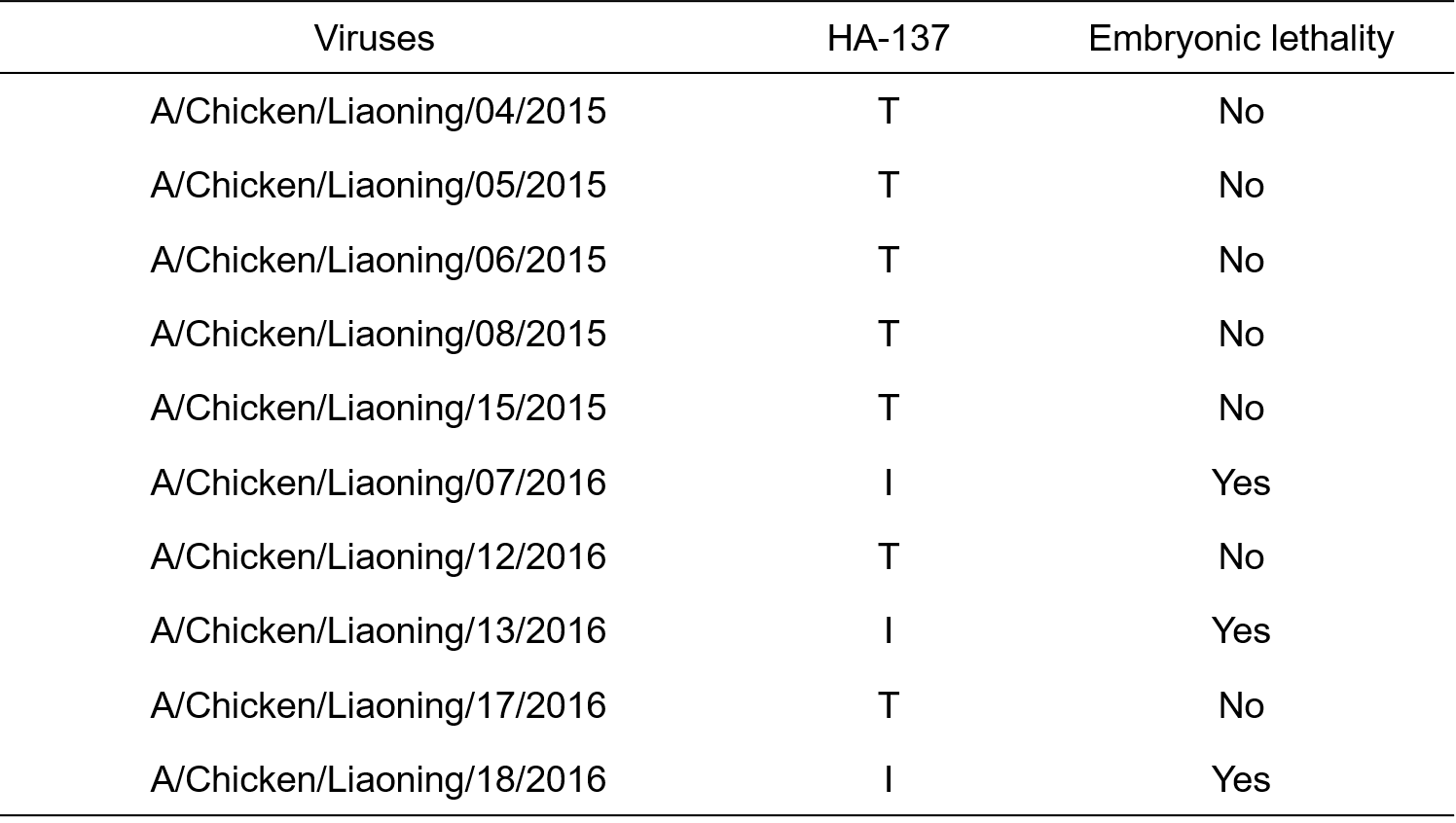


**Table S1** Virulence of H9N2 viruses in chicks embryo eggs

Supplement: Table S1.docx [file TEMI_A_2455597_SM8470.docx]

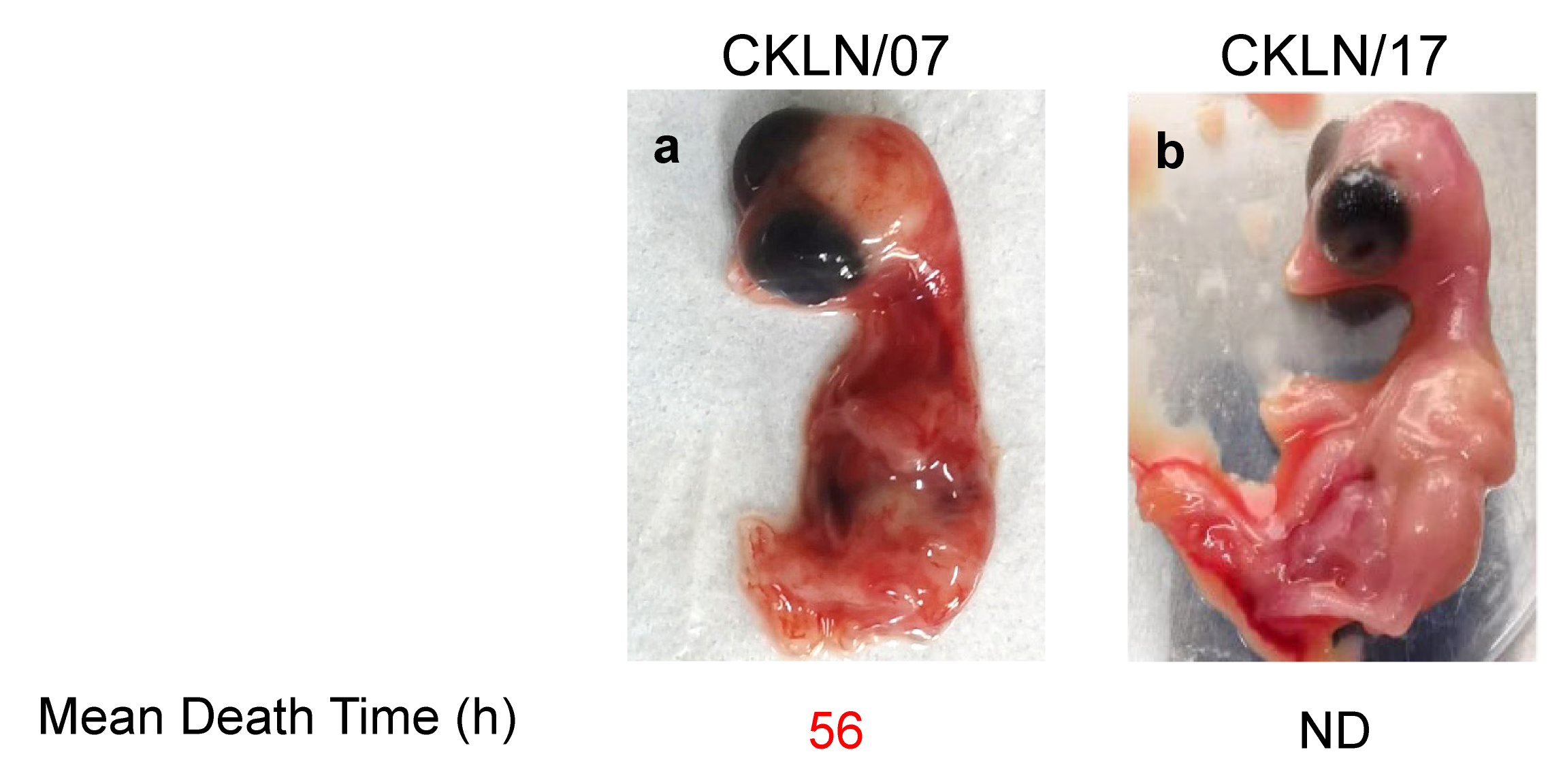

Supplement: Figure S2.tif [file TEMI_A_2455597_SM8468.tif]

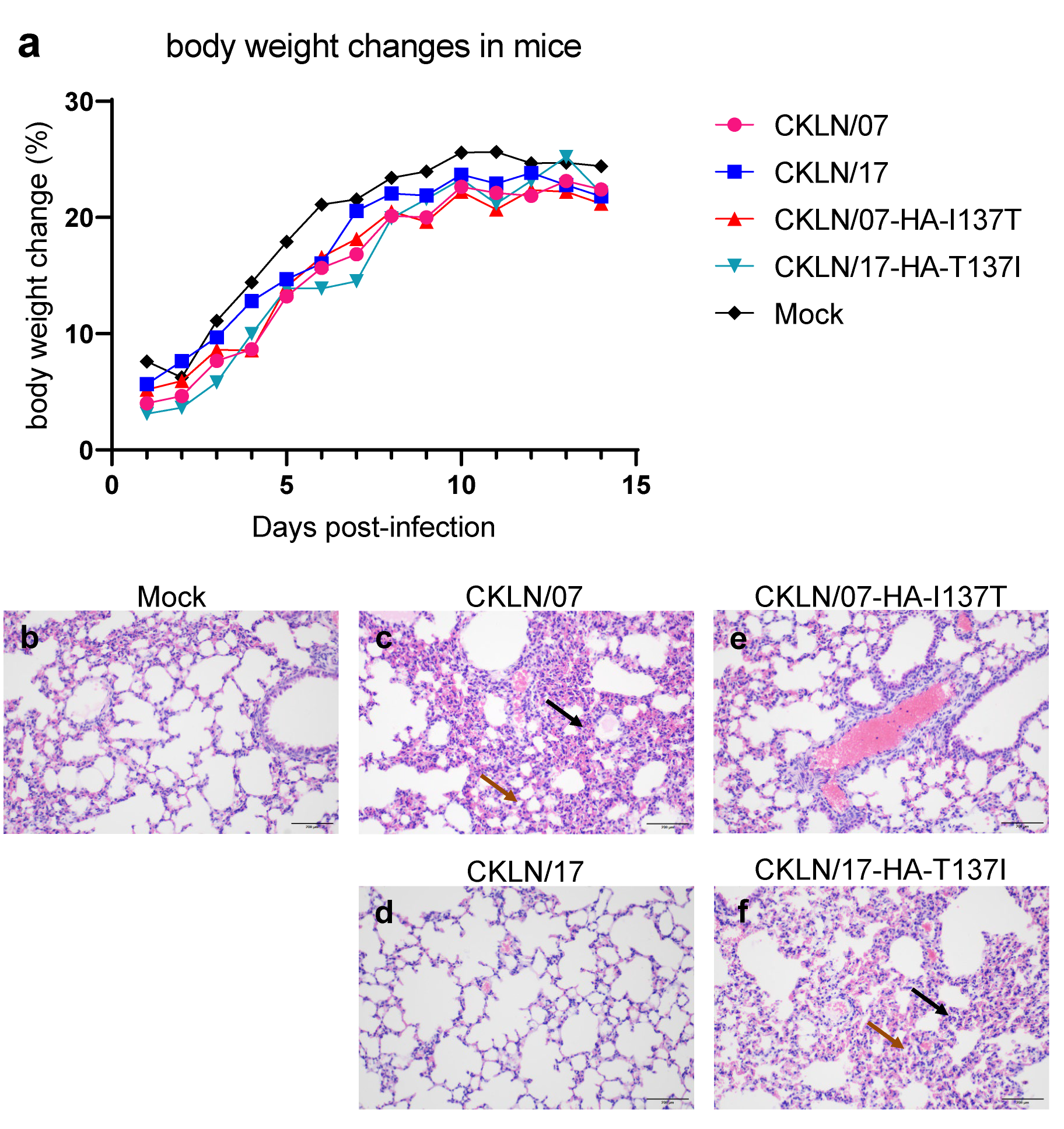

Supplement: Figure S4.tif [file TEMI_A_2455597_SM8465.tif]

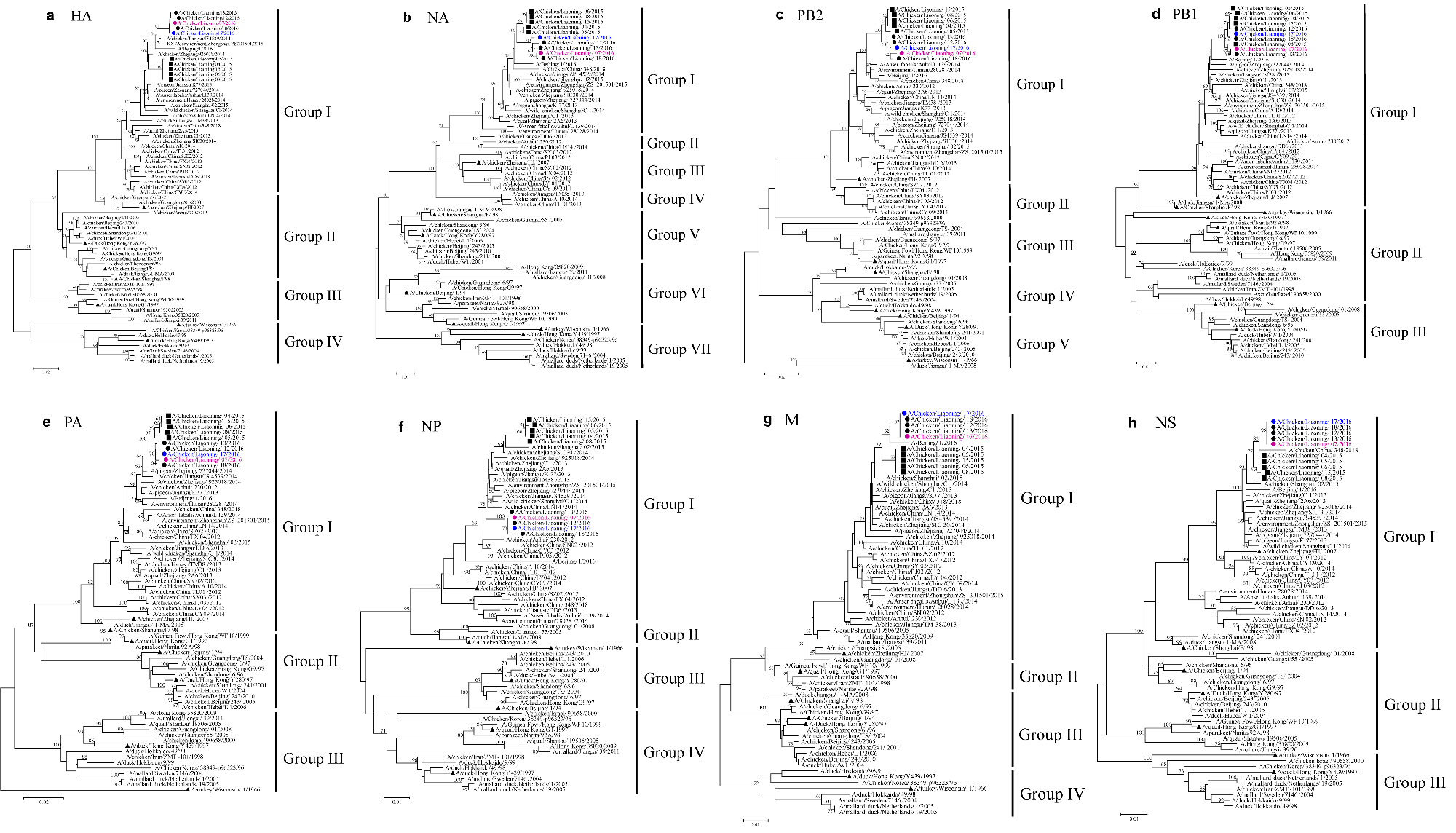


Figure s1

Supplement: Figure s1.docx [file TEMI_A_2455597_SM8464.docx]
