# Supplementary material for: Isoleucine at position 137 of haemagglutinin acts as a mammalian adaptation marker of H9N2 avian influenza virus
Source: Emerg Microbes Infect. 2025 Jan 16;14(1):2455597. doi: 10.1080/22221751.2025.2455597 (PMC11789229; doi:10.1080/22221751.2025.2455597)
Supplement: Supplementary materials.docx [file TEMI_A_2455597_SM8469.docx]

***Genomic sequencing and phylogenetic analyses***

Whole-genome sequencing and phylogenetic analyses were conducted on the isolated viruses. Viral RNA (vRNA) was extracted from virus-infected allantoic fluid using the TIAN amp Virus DNA/RNA Kit (TIANGEN Biotech, Beijing, China). Reverse transcription of viral RNA was performed using the Uni12 primer [1], followed by PCR using a set of primers (primer sequences available on request) of each gene segment. The PCR products were sequenced by Comate Bioscience Co.,Ltd.. Whole genomes sequences of the isolated viruses were assembled using Lasergene 7.1 software (DNAStar Inc., Madison, WI, USA) and upload to NCBI database, their gene bank access numbers are MW255942-MW255957 and PQ432337-PQ432400.

For phylogenetic analysis, we downloaded 59 reference viruses previously isolated from the GISAID EpiFlu database (https://platform.gisaid.org/). Phylogenetic tree was constructed using the distance-based neighbor-joining method with 1000 bootstrap replicates through the MEGA 7 (Pennsylvania State University, PA, USA). The tree's reliability was assessed through 1000 resampling tests. 95% sequence identity were used to group the genes in the phylogenetic trees.

***ELD_50_ and MLD_50_ assays***

The 50% egg lethal dose (ELD_50_) were determined by inoculating groups of five 9-day-old specific-pathogen-free (SPF) chick embryos (Harbin Veterinary Research Institute, Heilongjiang, China) with 10-fold serial dilutions containing 10^7.5^ to 10^0.5^ TCID_50_ of CKLN/07, 10^7.3^ to 10^0.3^ TCID_50_ of CKLN/17, 10^7.0^ to 10^0^ TCID_50_ of CKLN/07-HA-I137T and 10^7.2^ to 10^0.2^ TCID_50_ of CKLN/17-HA-T137I, in a volume of 100 µL.

The 50% mouse lethal dose (MLD_50_) were determined by inoculating groups of five 6-week-old female BALB/c mice (Changsheng Biotechnology, Liaoning, China) with10- fold serial dilutions containing of viruses, containing 10^7.2^ to 10^0.2^ TCID_50_ of CKLN/07, 10^7.0^ to 10^0^ TCID_50_ of CKLN/17, 10^6.7^ to 10^0.7^ TCID_50_ of CKLN/07-HA-I137T and 10^6.9^ to 10^0.9^ TCID_50_ of CKLN/07, in a volume of 50 µL.

The ELD_50_ and MLD_50_ values were calculated by using the Reed-Muench method [2] and expressed as median tissue culture infectious dose (TCID_50_).

***Pathological study***

Three 6-week-old female BALB/c mice (Changsheng Biotechnology, Liaoning, China) were intranasally infected with a 50-μL volume containing 10^6^ EID50 of CKLN/07, CKLN/17, CKLN/07-HA-I137T, CKLN/17-HA-T137I and PBS. On Day 3 post-inoculation, all mice were euthanized, and their lungs were collected and fixed overnight in 4% paraformaldehyde, followed by preparation of pathological sections using hematoxylin and eosin (H&E) staining.

***Viral replication in Chicks***

Six one-day-old SPF chicks (Harbin Veterinary Research Institute, Heilongjiang, China) were intranasally infected with 10^6^ EID_50_ of CKLN/07, CKLN/17, CKLN/07-HA-I137T and CKLN/17-HA-T137I in a volume of 100 μL. On Day 3 post-inoculation, three chicks were euthanized, and their brain, trachea, lung, spleen, pancreas, liver, intestine and kidney were collected and titrated with 10-day-old chick embryos. The remaining chicks were monitored until day 14 to assess clinical symptoms and mortality rates.

***Reference***

[1] Hoffmann E, Stech J, Guan Y, et al. Universal primer set for the full-length amplification of all influenza A viruses. Arch Virol. 2001;146(12):2275-89.

[2] Reed LJ, Muench H. A simple method of estimating fifty per cent endpoints. American Journal of Epidemiology. 1938;27(3):493-7.
