# Supplementary material for: Isoleucine at position 137 of haemagglutinin acts as a mammalian adaptation marker of H9N2 avian influenza virus
Source: Emerg Microbes Infect. 2025 Jan 16;14(1):2455597. doi: 10.1080/22221751.2025.2455597 (PMC11789229; doi:10.1080/22221751.2025.2455597)
Supplement: Table S2.docx [file TEMI_A_2455597_SM8467.docx]

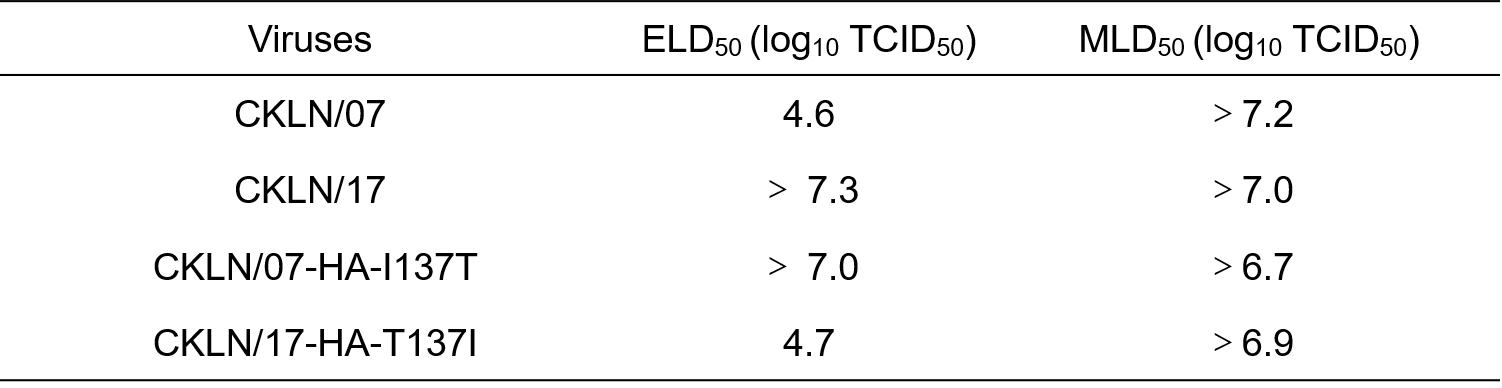


**Table S2** ELD_50_ and MLD_50_ of CKLN/07, CKLN/17 and the mutant viruses

Note: ELD_50_ and MLD_50_ expressed as median tissue culture infectious dose (TCID_50_)
