# Supplementary material for: Isoleucine at position 137 of haemagglutinin acts as a mammalian adaptation marker of H9N2 avian influenza virus
Source: Emerg Microbes Infect. 2025 Jan 16;14(1):2455597. doi: 10.1080/22221751.2025.2455597 (PMC11789229; doi:10.1080/22221751.2025.2455597)
Supplement: Table S3.docx [file TEMI_A_2455597_SM8466.docx]

**Table S3** The key amino acids of each segment of the isolates

|  | HA-155 | HA-160 | HA-183 | HA-187 | HA-189 | HA-190 | HA-208 | HA-212 | HA-226 | HA-227 | HA-228 | HA-246 |
| --- | --- | --- | --- | --- | --- | --- | --- | --- | --- | --- | --- | --- |
| A/Chicken/Liaoning/04/2015 | T | D | N | T | D | T | E | I | L | M | G | K |
| A/Chicken/Liaoning/05/2015 | T | D | N | T | D | T | E | I | L | M | G | K |
| A/Chicken/Liaoning/06/2015 | T | D | N | T | D | T | E | I | L | M | G | K |
| A/Chicken/Liaoning/08/2015 | T | D | N | T | D | T | E | I | L | M | G | K |
| A/Chicken/Liaoning/15/2015 | T | D | N | T | D | T | E | I | L | M | G | K |
| A/Chicken/Liaoning/07/2016 | T | E | N | A | T | T | E | I | L | M | G | K |
| A/Chicken/Liaoning/12/2016 | T | E | N | A | T | T | E | I | L | M | G | K |
| A/Chicken/Liaoning/13/2016 | T | E | N | A | T | T | E | I | L | M | G | K |
| A/Chicken/Liaoning/17/2016 | T | E | N | A | T | T | E | I | L | M | G | K |
| A/Chicken/Liaoning/18/2016 | T | E | N | A | T | T | E | I | L | M | G | K |
| References | [1] | [2] | [3] | [4] | [5] | [3, 6] | [2] | [6] | [3] | [2] | [7] | [2] |
|  | HA-316 | HA-363 | HA-375 | PB2-155 | PB2-588 | PB2-590 | PB2-591 | PB2-627 | PB2-685 | PB2-701 | PB1-368 | PA-49 |
| A/Chicken/Liaoning/04/2015 | A | M | D | S | V | G | Q | E | G | D | V | S |
| A/Chicken/Liaoning/05/2015 | A | M | D | S | V | G | Q | E | G | D | V | S |
| A/Chicken/Liaoning/06/2015 | A | M | D | S | V | G | Q | E | G | D | V | S |
| A/Chicken/Liaoning/08/2015 | A | M | D | S | V | G | Q | E | G | D | V | S |
| A/Chicken/Liaoning/15/2015 | A | M | D | S | V | G | Q | E | G | D | V | S |
| A/Chicken/Liaoning/07/2016 | A | M | D | S | V | G | Q | E | G | D | V | S |
| A/Chicken/Liaoning/12/2016 | A | M | D | S | V | G | Q | E | G | D | V | S |
| A/Chicken/Liaoning/13/2016 | A | M | D | S | V | G | Q | E | G | D | V | S |
| A/Chicken/Liaoning/17/2016 | A | M | D | S | V | G | Q | E | G | D | V | S |
| A/Chicken/Liaoning/18/2016 | A | M | D | S | V | G | Q | E | G | D | V | S |
| References | [8] | [3] | [9] | [10] | [3] | [3] | [11] | [12, 13] | [6] | [14] | [3] | [10] |
|  | PA-97 | PA-347 | PA-356 | NA-62-64 | NP-434 | M1-30 | M1-215 | M2-31 | NS1-103 | NS1-106 |  |  |
| A/Chicken/Liaoning/04/2015 | T | D | R | Deletion | E | D | A | N | L | I |  |  |
| A/Chicken/Liaoning/05/2015 | T | D | R | Deletion | E | D | A | N | L | I |  |  |
| A/Chicken/Liaoning/06/2015 | T | D | R | Deletion | E | D | A | N | L | I |  |  |
| A/Chicken/Liaoning/08/2015 | T | D | R | Deletion | E | D | A | N | L | I |  |  |
| A/Chicken/Liaoning/15/2015 | T | D | R | Deletion | E | D | A | N | L | I |  |  |
| A/Chicken/Liaoning/07/2016 | T | D | R | Deletion | E | D | A | N | L | I |  |  |
| A/Chicken/Liaoning/12/2016 | T | D | R | Deletion | E | D | A | N | L | I |  |  |
| A/Chicken/Liaoning/13/2016 | T | D | R | Deletion | E | D | A | N | L | I |  |  |
| A/Chicken/Liaoning/17/2016 | T | D | R | Deletion | E | D | A | N | L | I |  |  |
| A/Chicken/Liaoning/18/2016 | T | D | R | Deletion | E | D | A | N | L | I |  |  |
| References | [13] | [10] | [15] | [8] | [9] | [16] | [16] | [17] | [18] | [18] |  |  |

**References**

[1] Li X, Shi J, Guo J, et al. Genetics, receptor binding property, and transmissibility in mammals of naturally isolated H9N2 Avian Influenza viruses. PLoS Pathog. 2014;10(11):e1004508.

[2] Liu Y, Li S, Sun H, et al. Variation and Molecular Basis for Enhancement of Receptor Binding of H9N2 Avian Influenza Viruses in China Isolates. Front Microbiol. 2020;11:602124.

[3] Sun X, Belser JA, Maines TR. Adaptation of H9N2 Influenza Viruses to Mammalian Hosts: A Review of Molecular Markers. Viruses. 2020;12(5):541.

[4] Liu K, Guo Y, Zheng H, et al. Enhanced pathogenicity and transmissibility of H9N2 avian influenza virus in mammals by hemagglutinin mutations combined with PB2-627K. Virol Sin. 2023;38(1):47-55.

[5] Srinivasan K, Raman R, Jayaraman A, et al. Quantitative characterization of glycan-receptor binding of H9N2 influenza A virus hemagglutinin. PLoS One. 2013;8(4):e59550.

[6] Yang W, Lambertz RLO, Punyadarsaniya D, et al. Increased virulence of a PB2/HA mutant of an avian H9N2 influenza strain after three passages in porcine differentiated airway epithelial cells. Vet Microbiol. 2017;211:129-34.

[7] Vines A, Wells K, Matrosovich M, et al. The role of influenza A virus hemagglutinin residues 226 and 228 in receptor specificity and host range restriction. J Virol. 1998;72(9):7626-31.

[8] Sun Y, Tan Y, Wei K, et al. Amino acid 316 of hemagglutinin and the neuraminidase stalk length influence virulence of H9N2 influenza virus in chickens and mice. J Virol. 2013;87(5):2963-8.

[9] Sang X, Wang A, Ding J, et al. Adaptation of H9N2 AIV in guinea pigs enables efficient transmission by direct contact and inefficient transmission by respiratory droplets. Sci Rep. 2015;5:15928.

[10] Guo Y, Bai X, Liu Z, et al. Exploring the alternative virulence determinants PB2 S155N and PA S49Y/D347G that promote mammalian adaptation of the H9N2 avian influenza virus in mice. Vet Res. 2023;54(1):97.

[11] Wang C, Lee HH, Yang ZF, et al. PB2-Q591K Mutation Determines the Pathogenicity of Avian H9N2 Influenza Viruses for Mammalian Species. PLoS One. 2016;11(9):e0162163.

[12] Liang L, Jiang L, Li J, et al. Low Polymerase Activity Attributed to PA Drives the Acquisition of the PB2 E627K Mutation of H7N9 Avian Influenza Virus in Mammals. mBio. 2019;10(3):e01162-19.

[13] Cheng K, Yu Z, Chai H, et al. PB2-E627K and PA-T97I substitutions enhance polymerase activity and confer a virulent phenotype to an H6N1 avian influenza virus in mice. Virology. 2014;468-470:207-13.

[14] Li Z, Chen H, Jiao P, et al. Molecular basis of replication of duck H5N1 influenza viruses in a mammalian mouse model. J Virol. 2005;79(18):12058-64.

[15] Xu G, Zhang X, Gao W, et al. Prevailing PA Mutation K356R in Avian Influenza H9N2 Virus Increases Mammalian Replication and Pathogenicity. J Virol. 2016;90(18):8105-14.

[16] Fan S, Deng G, Song J, et al. Two amino acid residues in the matrix protein M1 contribute to the virulence difference of H5N1 avian influenza viruses in mice. Virology. 2009;384(1):28-32.

[17] Wang J, Wu Y, Ma C, et al. Structure and inhibition of the drug-resistant S31N mutant of the M2 ion channel of influenza A virus. Proc Natl Acad Sci U S A. 2013;110(4):1315-20.

[18] Dankar SK, Wang S, Ping J, et al. Influenza A virus NS1 gene mutations F103L and M106I increase replication and virulence. Virol J. 2011;8:13.
